# Transcriptome analysis and development of EST-SSR markers in *Anoectochilus emeiensis* (Orchidaceae)

**DOI:** 10.1371/journal.pone.0278551

**Published:** 2022-12-06

**Authors:** Song Lu

**Affiliations:** Sichuan Natural Resources Academy, Chengdu, Sichuan, China; Institute for Biological Research, University of Belgrade, SERBIA

## Abstract

*Anoectochilus emeiensis* K. Y. Lang, together with other *Anoectochilus* species, has long been used as the main source of many traditional Chinese medicines. Owing to the shortcomings of molecular markers, the study of the genetic diversity and medicinal component synthesis mechanism of the endemic *Anoectochilus* species has been delayed. In this study, I carried out a transcriptome analysis of *A*. *emeiensis*. A total of 78,381 unigenes were assembled from 64.2 million reads, and 47,541 (60.65%) unigenes were matched to known proteins in the public databases. Then, 9284 expressed sequence tag-derived simple sequence repeats (EST-SSRs) were identified, and the frequency of SSRs in the *A*. *emeiensis* transcriptome was 9.88%. Trinucleotide repeats (3699, 39.84%) were the most common type, followed by dinucleotide (3251, 35.02%) and mononucleotide (1750, 18.85%) repeats. Based on the SSR sequence, 6683 primer pairs were successfully designed, 40 primer pairs were randomly selected, and 10 primer pairs were identified as polymorphic loci from 186 individuals of *A*. *emeiensis*. The EST-SSR markers examined in this study will be informative for future population genetic studies of *A*. *emeiensis*.

## 1. Introduction

*Anoectochilus emeiensis* K. Y. Lang, an endemic ethnomedicinal plant, is distributed on Mount Omei, southwestern China. It is a traditional Chinese perennial herbal medicine, similar to other species of the genus *Anoectochilus* (Orchidaceae), including *A*. *formosanus*, *A*. *roxburghii*, and *A*. *koshunensis*. In Chinese folk, it was named “King Medicine” owing to its diverse pharmacological effects such as the remedy for diabetes, hypertension, fever, tumor, liver disease, snakebite, and lung disease [1]. *Anoectochilus* species are usually diploid, with 40 chromosomes [2]. The whole diploid genome DNA content has been found to be approximately 6.83 pg based on flow cytometry analysis [3]. Overexploitation and depletion have occurred with the constantly increasing demand for wild *A*. *emeiensis* and other *Anoectochilus* species for their medicinal properties and ornamental value. Currently, *A*. *emeiensis* is considered “critically endangered” by the China Species Red List because of its habitat degradation or loss, narrow distribution, few populations, and small population sizes [4].

The revelation of population structure, local adaptation, and differences in diversity between populations is vital for understanding the genetic variation within a species [5]. Previous studies on *A*. *emeiensis* and other *Anoectochilus* species have mainly focused on the evaluation of germplasm resources, seed breeding, artificial cultivation techniques, chemical composition, and pharmacological effects [1]. However, molecular research and wild population protection of *A*. *emeiensis* have fallen behind.

As the most popular markers in population genetic studies, simple sequence repeats have been increasingly used in fingerprinting, parentage analysis, genetic mapping, and genetic structure analysis because of their codominant and highly polymorphic nature in recent years [6–8]. In the past two decades, next-generation sequencing technology has been developed so quickly that not only model but also non-model organisms without sequenced genomes have been successfully sequenced for their high-throughput, accurate, and cost-effective advantages in transcriptome analysis [9–13]. Transcriptome sequencing is an efficient process for producing expressed sequence tags that are valuable for the development of molecular markers, gene annotation and discovery, and expression profiling [9, 11, 14]. Because of the lengthy and costly development phase and relatively low throughput, the development of single sequence repeats (SSRs) based on genome sequencing has not been as frequently adopted as transcriptome sequencing. Expressed sequence tag-derived SSR (EST-SSR) markers have been successfully developed by *de novo* transcriptome sequencing and assembly in many crops, such as barley, rice, and wheat [15–17], and in some Chinese herbal medicines, such as *Dendrobium officinale*, *Dysosma versipellis*, *Panax ginseng*, *Panax vietnamensis*, and *Siberian ginseng* [18–21]. To better protect wild germplasm resources, it is imperative to sequence the transcriptome of *A*. *emeiensis*. In the present study, the transcriptome of *A*. *emeiensis* leaves was sequenced using the Illumina HiSeq 2000 platform, and several EST-SSRs were derived. To our knowledge, this is the first systematic report of the complete transcriptome of *A*. *emeiensis*. The aims of this study were to (1) characterize and analyze the transcriptome of *A*. *emeiensis*, (2) determine the frequency and distribution of SSRs, (3) establish EST-SSR markers and examine the level of polymorphism, and (4) assess the genetic diversity of *A*. *emeiensis* among six populations.

## 2. Materials and methods

### 2.1. Plant materials, DNA, and RNA isolation

A total of 186 samples of *A*. *emeiensis* were collected from six natural populations in Mount Omei, Sichuan Province, China, at Longdongcun (LDC), Longdonghu (LDH), Daping (DP), Wuhe (WH), Jinzhulin (JZL), and Chuanzhusi (CZS) (August 8, 2015). Field research was approved by the Emeishan Ecological Environment Bureau, Sichuan Province, China. The collected fresh leaves of *A*. *emeiensis* had a height of 8–10 cm. After freezing in liquid nitrogen, all leaves were stored at −80 °C until DNA and total RNA extraction. Genomic DNA was isolated using DNA Plantzol (QIAGEN, Dusseldorf, Germany), following the manufacturer’s protocol. Voucher specimens were maintained in the herbarium of the Sichuan Institute of Natural Resources. Total RNA from ten different individuals was extracted using TRIzol reagent (Invitrogen Life Technologies, USA), following the manufacturer’s instructions. The quality and concentration of RNA were determined using a NanoDrop ND-1000 spectrophotometer (Thermo Scientific, Wilmington, DE, USA) and an Agilent 2100 Bioanalyzer (Agilent Technologies, Santa Clara, CA, USA). Equal proportions of high-quality RNA were pooled from each individual to construct a cDNA library.

### 2.2. Transcriptome sequencing, assembly, and annotation

Following the methods described by Liu et al. [10, 14, 22], a normalized cDNA library of *A*. *emeiensis* leaves was constructed. Briefly, the total RNA was collected and the poly(A) mRNA was purified with magnetic oligo(dT) beads. Then the mRNA was cleaved to short fragments by an RNA Fragmentation Kit (Ambion, Austin, TX), which were then used as templates to reverse-transcribe first-strand cDNA using random hexamer primers and reverse transcriptase (Invitrogen, Carlsbad, CA). Second-strand cDNA was synthesized in a reaction containing buffer, dNTPs, RNase H (Invitrogen) and DNA polymerase I (Qingkeweiye Biological Technology, Chengdu). The library was paired-end synthesized according to Illumina Genomic Sample Preparation Kit manufacturer’s instructions and sequenced using Illumina HiSeq 2000 (Illumina Inc., San Diego, CA, USA) at One Gene Company Limited, Hangzhou, China. Image analysis, base calling, and quality value calculations were performed using the Illumina GA Pipeline version 1.6. The raw Illumina sequencing data were then submitted to the NCBI Sequence Read Archive (accession number: SRR8138703) (SRA, http://www.ncbi.nlm.nih.gov/Traces/sra). Clean reads were obtained by removing those of low quality (more than 5% ambiguous bases “N”) and reads with more than 20% low-quality bases (value of ≤10) from raw reads using FastQC Version 0.11.5 (https://www.bioinformatics.babraham.ac.uk/projects/fastqc/). *De novo* assembly was performed using Trinity [12]. Generally, clean reads were combined to form longer fragments without Ns, termed contigs. The remaining isolated reads are described as singletons. Individual reads were then arranged back to the contigs using paired-end mapping. Subsequently, the order and distance between these contigs were determined from the same transcript. Finally, Trinity connected contigs and obtained sequences that could not be extended at either end, which were named as unigenes. These unigenes were further processed by sequence splicing and redundancy removal using TGICL version 2.1 to obtain nonredundant unigenes [23]. To intensively study the putative functions, the unigenes were compared using the BLASTX search (http://blast.ncbi.nlm.nih.gov/Blast.cgi) with a cut-off *E* value of 10^−5^ against protein databases such as NCBI nonredundant protein sequences (Nr), Swiss-Prot (http://www.expasy.ch/sprot/), the Kyoto Encyclopedia of Genes and Genomes (KEGG) pathway database (http://www.genome.jp/kegg), and the Clusters of Orthologous Groups (COG) database (http://www.ncbi.nlm.nih.gov/COG). To further annotate the unigenes, gene ontology (GO) was performed using the Blast2GO program (B2G; http://www.blast2go.com) [24].

### 2.3. Identification of EST-SSRs and primers design

SSR loci were detected using MIcroSAtellite software (MISA) (http://pgrc.ipkgatersleben.de/misa) [16]. The minimum number of repeats was six for dinucleotides, five for tri- and tetranucleotides, four for penta- and hexanucleotides, and twelve for mononucleotides. PRIMER version 3.0 [25] was used to design the primer pairs with the following settings: 18–28 bases (optimum 23 bp), product size of 80–160 bp, annealing temperature between 55 °C and 65 °C (optimum 60 °C), and CG content from 40% to 60% (optimum 50%). The potential primer pairs, hairpin structure, and occurrence of mismatches were checked using OLIGO (version 6.67; Molecular Biology Insights, Inc., Cascade, CO, USA). Finally, the specificity of the primer pairs was checked using BLAST against EST sequences.

### 2.4. EST-SSR analysis

The EST-SSR markers were initially tested for amplification using DNA from 24 *A*. *emeiensis* individuals to optimize the annealing temperature. In total, 40 SSRs were selected, and M13 (universal sequence-TGTAAAACGACGGCCAGT) [26] was added to the 5’ end of each forward primer of pairs and synthesized by Qingkeweiye Biological Technology (Chengdu) Co., Ltd. Polymerase chain reaction (PCR) amplification was performed using a thermal cycler GeneAmp PCR System 9700 (Applied Biosystems, Foster City, USA) in a 10 μl reaction mixture containing 1 μl of genomic DNA (50 ng), 0.25 unit *Taq* DNA polymerase (TaKaRa, Dalian, Liaoning, China), 1 × PCR buffer, 1 μl of 2.5 mM MgCl^2^, 1 μl of 2.5 mM dNTPs, 0.1 μl bovine serum albumin (TaKaRa), 0.5 μl of each 10 μM primer, and 0.5 μl of 10 μM fluorescent labeled M13 primer. The PCR amplification conditions were as follows: hot start at 95 °C for 5 min, followed by 35 cycles of denaturation at 94 °C for 45 s, annealing at various primer-specific temperatures for 45 s, extension at 72 °C for 1 min, and a final extension at 72 °C for 10 min. The PCR products were analyzed using a LI-COR 4300 DNA Analyzer (Gene Company Limited, USA), and their sizes were scored and compiled using the SAGA Generation 2 software (LI-COR).

### 2.5. Selective neutrality tests and population genetic analysis

The number of observed alleles (*N*_A_), observed (*H*_O_) and expected (*H*_E_) heterozygosity, and polymorphism information content (PIC) in each locus were calculated to test the polymorphism level using CERVUS version 3.0.3 [27]. For each population and locus of *A*. *emeiensis*, the diversity and inbreeding parameters, such as *N*_A_, *H*_E_, allelic richness (*R*_S_, standardized for the smallest number of individuals per unit using rarefaction), and the average inbreeding coefficient across all loci (*F*_IS_) were estimated using FSTAT version 2.9.3 [28].

## 3. Results

### 3 1. Assembly of *A*. *emeiensis* transcriptome data, functional annotation, and categorization of unigenes

In total, 10.7 Gb, 71,389,320 raw reads of 90 bp in length and 64,177,276 clean reads (89.90%) with 97.72% Q20 bases (base quality greater than 20) were generated. The total length of the reads was approximately 9.6 Gb (Table 1). The percentage of ambiguous bases and GC percentage for *A*. *emeiensis* were 0 and 47.70%, respectively. After TRINITY assembly, high-quality reads were assembled into 115,283 contigs with a mean length of 852 bp and an N50 length of 1375 bp. These contigs were then stitched into 78,381 unigenes with a mean length of 1002 bp and an N50 length of 1602 bp. Of the 78,381 unigenes with a total length of approximately 78.5 Mb, 33,002 unigenes (42.10%) had a length range of 200–500 bp, 35,462 unigenes (45.24%) had a length range of 501–1000 bp, and 9917 unigenes (12.65%) had a length of more than 2000 bp.

**Table 1:** Summary of the analysis of *A*. *emeiensis* transcriptomic data.

Number of raw reads	71,389,320
Number of high-quality reads	64,177,276
Number of clean nucleotides (nt)	9,626,591,400
Total length of contigs (bp)	98,217,609
Total length of unigenes (bp)	78,535,310
Number of contigs	115,283
Number of unigenes	78,381
Number of singletons	45,700
Total number of identified SSRs	9284
Number of SSR-containing sequences	7,742
Number of sequences containing more than one EST-SSR	1,195
Number of SSRs present in compound formation	791

Sequence similarity was determined using the BLAST algorithm. Of the 78,381 unigenes, 47,541 (60.65%) matched the known proteins in the public databases searched. That is 45,228 (57.70%), 34,547 (44.08%), 29,347 (34.77%), 27,793 (35.46%), 19,203 (24.50%), and 28,464 (36.31%) unigenes in the Nr, Nt, SwissProt, KEGG, COG, and GO databases, respectively. Furthermore, 80.8% of the Nr mapped unigenes showed high homologous matches to available plant sequences (*E* value <10^−15^), and 58.9% had significant conservation (*E* value <10^−45^). Regarding the similarity distribution, 86.5% of the Nr assembled sequences showed more than 40% similarity to known sequences, and 13.9% of the annotated sequences showed more than 80% similarity. For the species distribution, 16.4% (7395) annotated sequences were hit on *Vitis vinifera*, while 3.5–6.7% orthologous genes were distributed in *Amborella trichopoda*, *Amygdalus persica*, *Chaetochloa italica*, *Zea mays*, *Japanese rice*, and *Theobroma cacao*.

**Fig 1:**
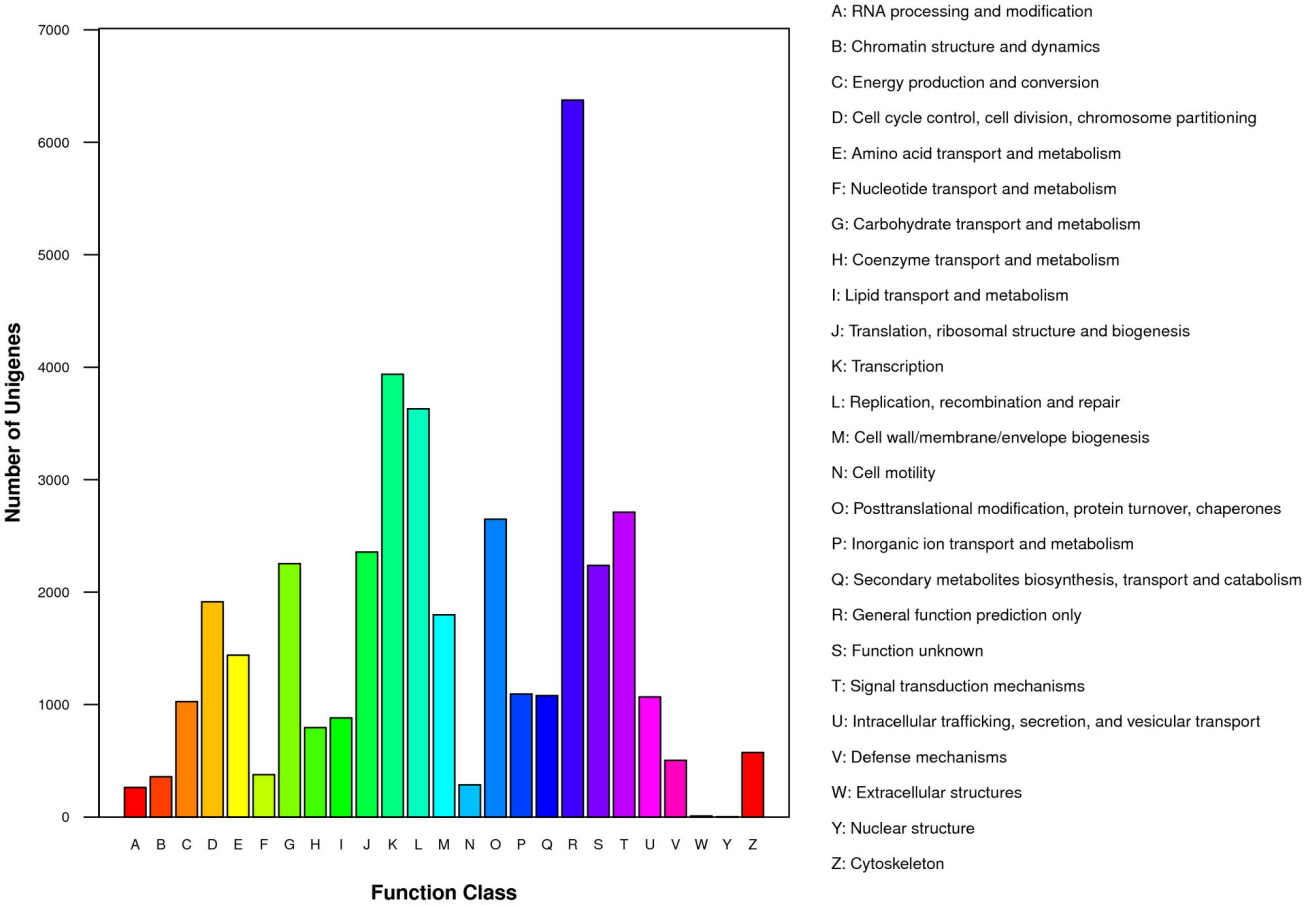
COG function classification by BLASTx with an E value threshold of 10^−^5 against COG databases.

A total of 19,203 unigenes were aligned to the COG database, and 39,622 matched unique sequences were clustered into 25 COG categories (Fig 1). The largest category among these classifications was the general function prediction only (6375, 16.09%), followed by transcription (3937, 9.94%); replication, recombination, and repair (3630, 9.16%); signal transduction mechanisms (2711, 6.84%); post-translational modification, protein turnover, and chaperones (2649, 6.69%); translation, ribosomal structure, and biogenesis (2357, 5.95%); and carbohydrate transport and metabolism (2253, 5.69%). Approximately 2238 (5.65%) unigenes were poorly characterized as function unknown. Extracellular and nuclear structures contained the fewest unigenes.

**Fig 2:**
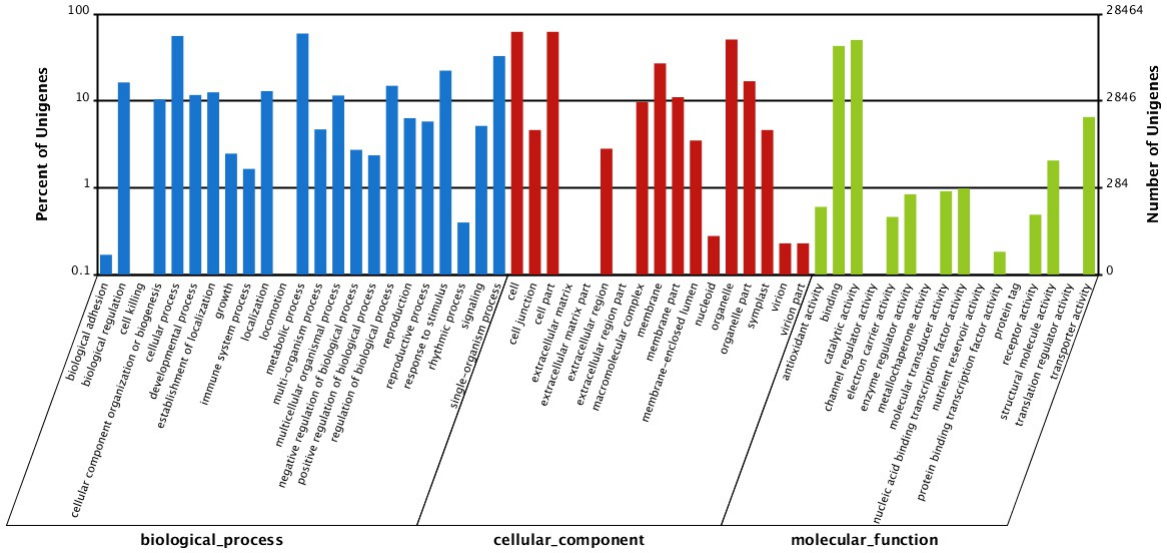
GO classification for *A*. *emeiensis*. by BLASTx with an E value threshold of 10^−^5 against the Nr database.

Using the Blast2GO software, 28,464 (41.63%) matched unigenes were assigned to three main categories: biological process (83,272, 44.64%), cellular component (73,002, 39.14%), and molecular function (30,251, 16.22%) (Fig 2). Fifty-six subcategories were further subdivided into GO terms. The three major sub-categories of the biological process were ‘‘metabolic process” (16,960, 20.37%), ‘‘cellular process” (15,899, 19.09%), and ‘‘single-organism process” (9339, 11.22%). In terms of cellular components, ‘‘cell” (17,746, 24.31%), ‘‘cell part” (17,746, 24.31%), ‘‘organelle” (14,474, 19.83%), and ‘‘membrane” (7683, 10.52%) represented the most common terms. Catalytic activity (14,298, 47.26%), binding (12,202, 40.33%), and transporter activity (1853, 6.12%) were the dominant molecular functions. To further explore the biological pathways and gene interactions of *A*. *emeiensis*, a BLASTX search was performed against the KEGG database: 27,793 (35.46%) of the 78,381 unigenes were grouped into 128 KEGG pathways. Of these pathways, metabolic pathways (7072, 25.45%) were the most representative, followed by biosynthesis of secondary metabolites (3146, 11.32%), endocytosis (1482, 5.33%), and glycerophospholipid metabolism (1409, 5.07%). Besides, pathways related to terpenoid backbone (265, 0.95%), flavonoid (211, 0.76%), flavone and flavonol (137, 0.49%), steroid (136, 0.49%), isoflavonoid (80, 0.29%), and monoterpenoid (15, 0.05%) biosynthesis were also represented by unigenes. These pathways may be conducive to research on the metabolism of active ingredients in *A*. *emeiensis*.

### 3.2. SSRs identification and characterization

**Fig 3:**
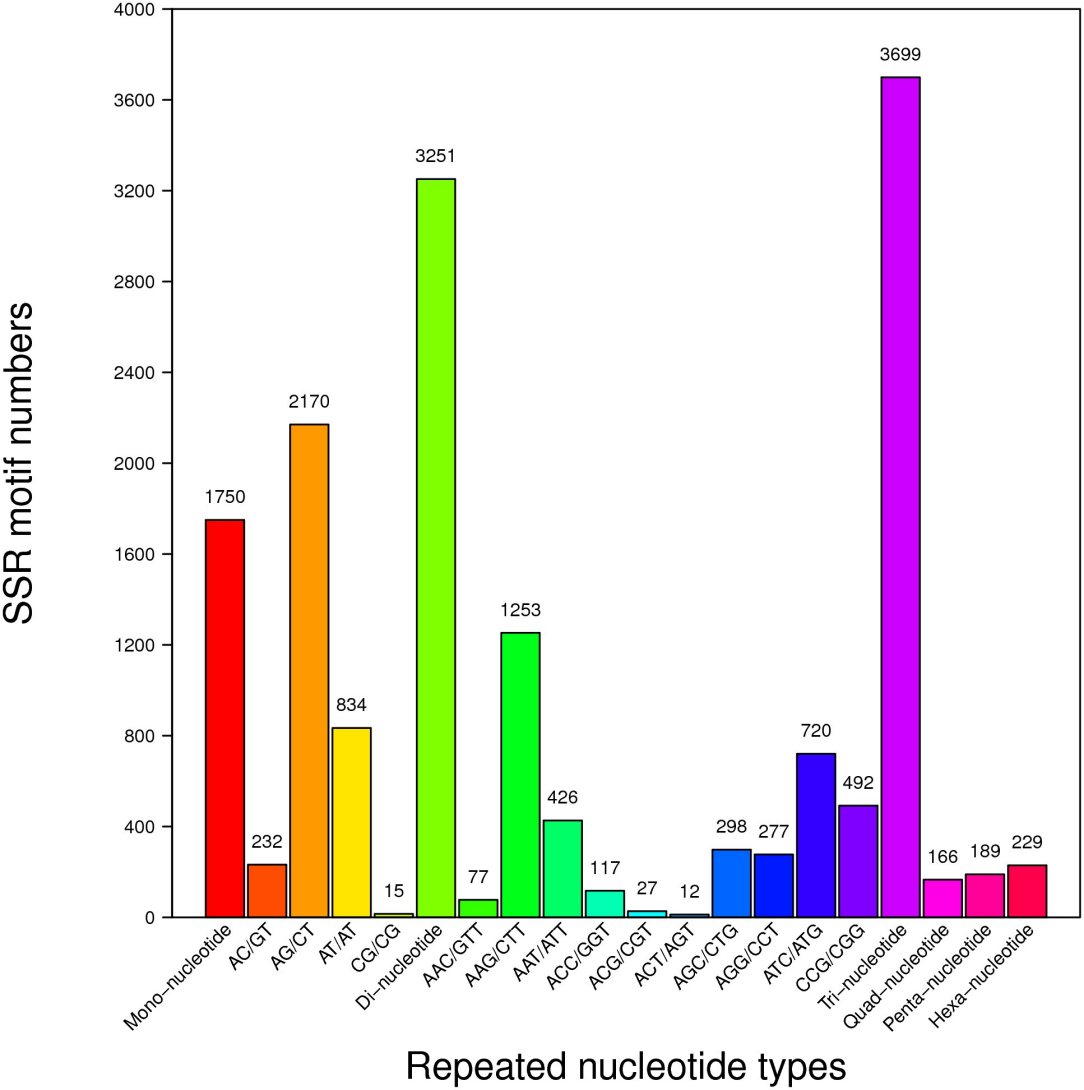
The SSRs repeated nucleotide types for *A*. *emeiensis*.

The results of the MISA search showed that 7742 unigenes contained 9284 EST-SSRs. There were 1195 unigenes containing more than one EST-SSR locus, and 678 SSRs were present during compound formation. The frequency of SSRs in the *A*. *emeiensis* transcriptome was 9.88%. Trinucleotide repeats (3699, 39.84%) were the most common type, followed by dinucleotides (3251, 35.02%), mononucleotides (1750, 18.85%), hexanucleotides (229, 2.47%), pentanucleotides (189, 2.04%), and tetranucleotides (166, 1.79%). In total, five tandem SSRs (22.27%) were the most common, followed by six (21.85%), seven (12.26%), and eight (8.61%). Subsequently, 355 different types of motifs were identified. Of the 3251 dinucleotide repeats, AG/CT was the most dominant motif (2170, 23.37%), followed by AT/AT (834, 8.98%), AC/GT (232, 2.50%), and CG/CG (15, 0.16%). In the 3699 trinucleotide repeats, the most abundant repeat motifs were AAG/CTT (1253, 13.50%), followed by ATC/ATG (720, 7.76%), CCG/CGG (492, 5.30%), AAT/ATT (426, 4.59%), AGC/CTG (298, 3.21%), AGG/CCT (277, 2.98%), ACC/GGT (117, 1.26%), AAC/GTT (77, 0.83%), ACG/CGT (27, 0.30%), and ACT/AGT (12, 0.13%) (Fig 3). The 14 repeat motifs mentioned above constituted 74.86% of the total, whereas the remaining 341 types constituted 25.14%.

### 3.3. Polymorphism of EST-SSR markers and population genetic diversity

A total of 6683 primer pairs were successfully designed, with amplicons ranging from 80 to 240. Subsequently, 40 primer pairs were randomly selected. In the initial screening of 24 individuals from the six populations, 10 primer pairs were polymorphic. These were used to test the polymorphism in 186 individuals from the six populations. A total of 53 alleles were detected at ten loci. The number of alleles (*N*_A_) ranged from 3 to 8, with an average of 5.3. The observed heterozygosity (*H*_*O*_) varied from 0.237 to 0.505 with a mean value of 0.39. The expected heterozygosity (*H*_E_) ranged from 0.238 to 0.799 with an average of 0.560. The PIC values fluctuated from 0.217 to 0.767, with a mean of 0.514 (Table 2).

**Table 2:** Ten EST-SSR markers developed in *A*. *emeiensis* and characteristics of each primer pairs.

Locus	Primer pair (5′-3′)	Repeat motif	Size range(bp)	*N*_A_	*H*_O_	*H*_E_	PIC	Tm (°C)
U1017	F:CACCAAAAGAGACTCATCTCACC R:TTGCCATTTATTGTCAAATTGCT	(CAT)_8_	156–177	8	0.446	0.799	0.767	60
U42760	F:CATCTTATCCTTGCTCTCCTCAA R:TGTTATCAGTGGGCTGAAACTG	(CGC)_7_	157–169	5	0.269	0.344	0.314	56
U32259	F:CTCTTCATCGATGTCCAAAGACT R:CTTCGTCTGGAACTTATTGGGTT	(TTC)_6_	123–135	5	0.505	0.729	0.678	58
CL1528	F:TCTACACCCATTCAAAGAAGCAT R:CAATCGGCATCTATTTTTACTCG	(CTGC)_5_	154–178	7	0.481	0.597	0.542	60
U28269	F:AGTTCCCTGCTTTATTTTGTTCC R:AAAAGGAGTTGATGCCTAGTGTG	(CT)_7_	156–166	5	0.359	0.695	0.636	58
U1140	F:AGCAAGGTAATAGTTGTGCCAAA R:TCCCTTCCCTCAAATTCTTATTC	(GA)_6_	157–165	5	0.337	0.518	0.470	58
CL1364	F:ACCTTTTTCCATTAATCTGGGAA R:CAACTCTCTCCGTCAAGTCAAAT	(GT)_6_	149–158	3	0.328	0.387	0.353	60
U7123	F:ATCTTGAAGTTCGATGCAGAAAG R:AACCCCTTTATGTATTCTTTGCAG	(AAG)_7_	113–128	5	0.237	0.238	0.217	56
U30086	F:CCCCATAAGTAATTGTTTTGCAT R:GACAGAGTTTGTGTGAGAGAGGG	(TG)_6_	155–158	4	0.481	0.678	0.629	56
CL3430	F:ATAACTTGGGGAAGCTGTGAAGT R:TGATTTGAAGTAAATGGCACAGA	(GA)_6_	153–168	6	0.457	0.612	0.531	56

*N*_A_ = number of alleles per locus; *H*_O_ = observed heterozygosity; *H*_E_ = expected heterozygosity; PIC = polymorphism information content; Tm = annealing temperature.

In each population, the mean number of alleles (*N*_A_) across the 10 surveyed SSR markers varied from 2.6 to 4.8, and the LDC population was the highest. The average allelic richness (*R*_S_) values ranged from 2.6 to 3.153, and the LDC population was also the highest. The mean expected heterozygosity (*H*_E_) was 0.0211–0.7134. The mean *F*_IS_ fluctuated from −0.058 to 0.332, and the value in the LDH population was the highest (Table 3).

**Table 3:** Characteristics of the 10 EST-SSRs in each population of *A*. *emeiensis*.

Locus	Longdongcun (LDC, *N* = 83)				Daping (DP, *N* = 45)				Longdonghu (LDH, *N* = 27)			
	*N*_A_	*R*_S_	*H*_E_	*F*_IS_	*N*_A_	*R*_S_	*H*_E_	*F*_IS_	*N*_A_	*R*_S_	*H*_E_	*F*_IS_
U1017	8	4.654	0	0.455	7	4.787	0.1312ns	0.201	6	4.207	0	0.472
U42760	5	2.548	0.2463ns	0.138	4	2.296	0.1382ns	0.275	3	2.082	0.0026	0.566
U32259	4	3.587	0.0004	0.290	5	3.713	0.0022	0.268	4	3.808	0.0003	0.417
CL1528	5	3.230	0.0706	0.159	6	3.131	0.0094	0.102	5	3.302	0.0055	0.445
U28269	5	3.626	0	0.467	4	3.443	0	0.436	4	2.885	0.0001	0.652
U1140	5	2.732	0	0.536	5	3.121	0.0213	0.284	4	3.159	0.4490	0.160
CL1364	3	2.436	0.0666	0.161	3	2.192	0.0258	0.266	3	2.486	1	-0.112
U7123	4	1.975	0.0524	0.172	4	1.848	1	-0.090	2	1.466	1	-0.040
U30086	4	3.496	0	0.291	4	3.456	0.0001	0.238	4	3.561	0.0002	0.533
CL3430	5	3.243	0.0003	0.313	4	2.903	0.5137	0.052	3	2.335	0.0192	0.231
Mean	4.8	3.153	0.0211	0.298	4.6	3.089	0.1966	0.203	3.8	2.929	0.2477	0.332
Locus	Jinzhulin (JZL, *N* = 19)				Chuanzhusi (CZS, *N* = 7)				Wuhe (WH, *N* = 5)			
	*N*_A_	*R*_S_	*H*_E_	*F*_IS_	*N*_A_	*R*_S_	*H*_E_	*F*_IS_	*N*_A_	*R*_S_	*H*_E_	*F*_IS_
U1017	5	4.353	0	0.596	4	3.867	0.0077	0.636	3	3.000	0.0159	1.000
U42760	2	1.462	1.0000ns	-0.029	2	1.934	1.0000ns	-0.091	2	2.000	1.0000ns	-0.143
U32259	5	3.959	0.0148ns	0.371	4	3.648	0.3606ns	0.000	3	3.000	0.619ns	0.360
CL1528	4	3.292	0.2470	0.225	4	3.647	0.2727	-0.500	2	2.000	-	0.000
U28269	4	3.405	0.1281	0.253	1	1.000	-	-	3	3.000	0.3333	0.238
U1140	4	2.918	0.2648	0.289	2	1.934	1	-0.091	3	3.000	1	-0.200
CL1364	3	2.185	0.0071	0.636	3	2.868	1	-0.200	2	2.000	1	-0.333
U7123	2	1.936	0.5384	-0.241	2	1.989	1	-0.200	2	2.000	1	-0.333
U30086	4	3.484	0.4428	0.038	3	2.923	1	-0.021	3	3.000	0.1111	0.143
CL3430	3	2.235	1	-0.087	1	1.000	-	-	3	3.000	0.3651	0.407
Mean	3.6	2.923	0.3285	0.205	2.6	2.481	0.7134	-0.058	2.6	2.6	0.5465	0.114

*N*_A_ = number of detected alleles per locus; *R*_S_ = allelic richness; *H*_E_ = expected heterozygosity; *F*_IS_ = inbreeding coefficient; n.s = not significant.

## 4. Discussion

### 4.1. Characterization of the *A*. *emeiensis* transcriptome

Previously, no ESTs of *A*. *emeiensis* were available in public databases. In this study, 78,381 unigenes were obtained from *A*. *emeiensis* leaves with a mean length of 1002 bp, which were longer than those of previous transcriptome studies of *A*. *formosanus* (mean length 679 bp), *Dendrobium officinale* (mean length 742 bp), *Panax ginseng* (mean length 469 bp), and *Siberian ginseng* (mean length 785 bp), but lower than those of *Panax vietnamensis* (mean length 1304 bp) [18–21, 29]. The number of unigenes assembled in *A*. *emeiensis* was lower than that reported for *A*. *formosanus* (173,513), *Panax ginseng* (178,145), and *Panax vietnamensis* (126,758), but higher than that in *Dendrobium officinale* (36,407) and *Dysosma versipellis* (44,855) [7]. Approximately 60.65% (47,541) unigenes were successfully annotated in public databases (Nr, Nt, Swiss-Prot, COG, GO, and KEGG). Compared with previous studies, the number of annotated unigenes in *A*. *emeiensis* was greater than that in *Dendrobium officinale* (25,473) but lower than those in *A*. *formosanus* (128,054), *Panax ginseng* (94,535), *Panax vietnamensis* (85,214), and *Siberian ginseng* (48,300). The major chemical constituents of *A*. *emeiensis* are polysaccharides, flavonoids, glycosides, steroids, and triterpenes et al. [1]. Unigenes sorted to these constituent biosynthesis pathways had been annotated as carbon metabolism, O-glycan and N-glycan biosynthesis, flavonoid, flavone and flavonol biosynthesis, terpenoid backbone biosynthesis, and mono-, di-, sesqui-, and triterpenoid biosynthesis.

In planting, the quality of *Anoectochilus* is mostly evaluated by the content of flavonoids, such as rutin, quercetin, isorhamnetin, and kaempferol [30, 31]. Flavonoids are important pharmacodynamic components in *Anoectochilus*. Flavonoid biosynthesis is very complex, and there are many intersection points in the synthetic pathway. According to the KEGG annotation and the species of flavonoids separated and purified from *Anoectochilus*, the key enzymes in the flavonoid synthesis pathway include chalcone synthase, chalcone isomerase, naringenin 3-dioxygenase, flavonol synthase, flavonoid 3’-hydroxylase, flavone O-methyltransferase, flavonol 3-O-glucosyltransferase, and flavonol-3-O-glucoside L-rhamnosyltransferase. These enzymes play key roles in the metabolic pathways from cinnamoyl-CoA, naringenin chalcone, naringenin, dihydrokaempferol, and kaempferol, to quercetin, isorhamnetin, isoquercetin, rutin, and narcissoside [32]. Many components are both intermediates and important pharmacodynamic substances such as kaempferol, quercetin, isoquercetin, and rutin. *A*. *emeiensis*, together with other species of *Anoectochilus*, have been simultaneously studied for novel gene discovery and exploration of their medicinal mechanism.

Furthermore, 4.47% unigenes were annotated to the plant-pathogen interaction pathway, most of which belonged to environmental adaptation groups. *Anoectochilus* plants are symbiotic with fungi and gain nutrition from the environment. These genes will help reveal how tiny exalbuminous seeds can survive under extreme conditions.

### 4.2. Frequency and distribution of EST-SSRs

A total of 9284 EST-SSRs were mined and distributed among 7742 unigenes. The SSR frequency in the *A*. *emeiensis* transcriptome (9.88%) was higher than that in maize (1.5%) and rice (4.7%) but lower than that in *A*. *formosanus* (12.21%) and *Neolitsea sericea* (16.25%), and it was the same as that in many dicot species that fall in this range of values (2.65–16.82%) [7, 29, 33]. The variable results may be due to different SSR mining software and criteria [33].

In *A*. *emeiensis*, the SSR motif types were not evenly distributed. Trinucleotide repeats (39.84%) were the most frequent type, followed by dinucleotides (35.02%) and mononucleotides (18.85%). The hexa-, penta-, and tetranucleotide repeat types were much rarer (2.47, 2.04, and 1.79%, respectively). The present result was congruent with earlier suggestions that trinucleotide repeats are the main EST-SSR type in both mono- and dicots [7, 34–37]. This is in contrast to many conclusions that dinucleotide repeats are the dominant repeats [33]. Also, this result is different from *A*. *formosanus*, in which mononucleotides have been determined to be the most common type (61.93%) [31]. The predominant trimeric SSRs may be attributed to the suppression of non-trimeric SSRs in coding regions [32]. In *A*. *emeiensis*, AAG/CTT (13.50%) and AG/CT (23.37%) motifs were the most common tri- and dinucleotide repeats, and CCG/CGG (5.30%) was also frequent. These results are congruent with those of previous studies on *Arabidopsis*, soybean, cucumber, sesame, cocoa, and caster bean, which also suggested that the trinucleotide AAG motif may be prominent in dicots [33, 38].

### 4.3. Polymorphism of EST-SSR markers and population genetic diversity and structure

In total, 53 alleles were detected across 186 *A*. *emeiensis* individuals from six populations with 10 polymorphic EST-SSR markers (Table 2). The *N*_A_ values across these markers ranged from 3 to 8 with a mean of 5.3, which were higher than those of *Dysosma versipellis* and *D*. *pleiantha*, but lower than that of *Neolitsea sericea* [7, 14]. The average PIC value was 0.514 for the ten developed loci. The PIC is used to assess the genetic information level. The results of this study showed that the PIC value fell at a medium level and showed high polymorphism.

After years of investigation, I found that there were two reproductive modes in the wild *A*. *emeiensis* community: asexual reproduction and insect pollination. Perennial rhizomes with induced *A*. *emeiensis* buds can grow to more than 20 cm. The new shoots grow completely from the succulent stem nodes. Although wild populations lack pollinating insects, *A*. *emeiensis* can occasionally be pollinated by insects. These factors contribute to the unique genetic structure of *A*. *emeiensis*.

Typical habitat fragmentation occurs in wild populations of *A*. *emeiensis*. Owing to the construction of roads, parking lots, and hotels, the distribution area has been isolated into several groups. Wild *A*. *emeiensis* was concentrated in the LDC, DP, LDH, and JZL populations. Allelic richness (*Rs*) was positively correlated with population size (Table 3). Almost all of the detected alleles (*N*_A_) declined in the small populations (Table 3). For example, in the LDC population, the *N*_A_ of U1017 was 8 but declined to 4 in CZS and 3 in WH. Thus, four and five alleles of U1017 were lost in the CZS and WH populations, respectively. The lowest allele frequency of U1017 was 174. However, this allele was lost in the JZL, CZS, and WH populations (S1 Table). The results showed that when the population becomes smaller owing to habitat fragmentation, the number of alleles in the remaining population mainly depends on the size of the population and the allele frequency distribution [39, 40]. Alleles with lower frequencies will be lost.

In each population, inbreeding was prominent among the individuals (Table 3): 83.33% of the mean inbreeding coefficient (*F*_IS_) was positive, providing evidence for this. Inbreeding increases homozygosity and reduces heterozygosity [41]. In the continuous inbreeding population, the frequency of heterozygotes tended to be zero. This was proven by the fact that *H*_E_ of many alleles in four larger populations (LDC, DP, LDH, and JZL) was 0. Lacy and Lindenmayer (1995) found that when the population is divided into different small parts, heterozygosity and allelic diversity decrease rapidly [42]. The results of this study support this conclusion.

Overall, the EST-SSRs derived from this study are of effective quality in *A*. *emeiensis*. These markers will aid in investigating the genetic structure of wild populations, and the generated database will help us understand the molecular synthesis mechanisms of medicinal components in *A*. *emeiensis*.

## 5. Conclusions

In this study, I performed the first transcriptome analysis of *A*. *emeiensis* leaves using the Illumina HiSeq 2000 sequencing system. A total of 78,381 unigenes were assembled from 64.2 million reads, and 47,541 (60.65%) unigenes were matched to known proteins in public databases. Then, 9284 EST-SSRs were identified, and the frequency of SSRs in the *A*. *emeiensis* transcriptome was 9.88%. Trinucleotide repeats (3699, 39.84%) were the most common type, followed by dinucleotide (3251, 35.02%) and mononucleotide (1750, 18.85%) repeats. Based on the SSR sequence, 6683 primer pairs were successfully designed, 40 primer pairs were randomly selected, and 10 primer pairs were identified as polymorphic loci from 186 individuals of *A*. *emeiensis*. The EST-SSR markers examined in this study will be informative for future population genetic studies of *A*. *emeiensis*.

## Supporting information

S1 Table Raw allele scoring table based on the 10 EST-SSRs in each population of *A*. *emeiensis*. (XLSX)
